# PNPLA3 Expression Is Related to Liver Steatosis in Morbidly Obese Women with Non-Alcoholic Fatty Liver Disease

**DOI:** 10.3390/ijms17050630

**Published:** 2016-04-27

**Authors:** Gemma Aragonès, Teresa Auguet, Sandra Armengol, Alba Berlanga, Esther Guiu-Jurado, Carmen Aguilar, Salomé Martínez, Fátima Sabench, José Antonio Porras, Maikel Daniel Ruiz, Mercé Hernández, Joan Josep Sirvent, Daniel Del Castillo, Cristóbal Richart

**Affiliations:** 1 Group de Recerca GEMMAIR (AGAUR)-Medicina Aplicada, Institut Investigació Sanitària Pere Virgili (IISPV), Departament de Medicina i Cirurgia, Universitat Rovira i Virgili (URV), 43007 Tarragona, Spain; gemma.aragones@iispv.cat (G.A.); tauguet.hj23.ics@gencat.cat (T.A.); sandra.armengol@urv.cat (S.A.); alba.berlanga@urv.cat (A.B.); esther.guiu@urv.cat (E.G.-J.); caguilar.hj23.ics@gencat.cat (C.A.); 2 Servei Medicina Interna, Hospital Universitari Joan XXIII Tarragona, Mallafré Guasch, 4, 43007 Tarragona, Spain; aporras.hj23.ics@gencat.cat (J.A.P.); drgorrin@yahoo.es (M.D.R.); 3 Servei Anatomia Patològica, Hospital Universitari Joan XXIII Tarragona, Mallafré Guasch, 4, 43007 Tarragona, Spain; mgonzalez.hj23.ics@gencat.cat (S.M.); jsirvent.hj23.ics@gencat.cat (J.J.S.); 4 Servei de Cirurgia, Hospital Sant Joan de Reus, Departament de Medicina i Cirurgia, Universitat Rovira i Virgili (URV), IISPV, Avinguda Doctor Josep Laporte, 2, 43204 Tarragona, Spain; fatima.sabench@urv.cat (F.S.); mhernandezg@grupsagessa.com (M.H.); ddelcastillo@grupsagessa.com (D.D.C.)

**Keywords:** PNPLA3, morbid obesity, non-alcoholic fatty liver disease, simple steatosis, fatty acid metabolism, non-alcoholic steatohepatitis

## Abstract

Recent reports suggest a role for the Patatin-like phospholipase domain-containing protein 3 (PNPLA3) in the pathology of non-alcoholic fatty liver disease (NAFLD). Lipid deposition in the liver seems to be a critical process in the pathogenesis of NAFLD. The aim of the present work was to evaluate the association between the liver *PNPLA3* expression, key genes of lipid metabolism, and the presence of NAFLD in morbidly obese women. We used real-time polymerase chain reaction (PCR) analysis to analyze the hepatic expression of *PNPLA3* and lipid metabolism-related genes in 55 morbidly obese subjects with normal liver histology (NL, *n* = 18), simple steatosis (SS, *n* = 20), and non-alcoholic steatohepatitis (NASH, *n* = 17). Liver biopsies were collected during bariatric surgery. We observed that liver *PNPLA3* expression was increased in NAFLD than in NL. It was also upregulated in SS than in NL. Interestingly, we found that the expression of *PNPLA3* was significantly higher in severe than mild SS group. In addition, the expression of the transcription factors *LXRα*, *PPARα*, and *SREBP2* was positively correlated with *PNPLA3* liver expression. Regarding rs738409 polymorphism, GG genotype was positive correlated with the presence of NASH. In conclusion, our results show that PNPLA3 could be related to lipid accumulation in liver, mainly in the development and progression of simple steatosis.

## 1. Introduction

Non-alcoholic fatty liver disease (NAFLD), the most common liver disease in Western countries, is characterized by the accumulation of excess triglycerides (TG) in hepatocytes and is associated with or anticipates the metabolic syndrome and its individual features, including visceral obesity, hyperlipidemia, and type 2 diabetes mellitus (T2DM) [1]. NAFLD includes a range of diseases from simple fatty infiltration (simple steatosis (SS)), fat accumulation, and inflammation (non-alcoholic steatohepatitis (NASH)) to liver fibrosis/cirrhosis [2]. General prevalence of NAFLD is 25.24%, with the highest prevalence in the Middle East and South America. This prevalence is particularly high in obese adults (80%–90%), patients with T2DM (30%–50%), and up to 90% in patients with hyperlipidemia [3]. NAFLD is usually diagnosed by abdominal ultrasonography in subjects without any apparent liver alteration who do not consume excessive alcohol [4]. Some studies have shown that insulin resistance (IR) promotes not only the recruitment of free fatty acids (FAs) in liver from the serum pool, but also the accumulation of intrahepatic FA, which indicates that IR is, among other mechanisms, crucial to the pathogenesis of NAFLD/NASH. In this regard, some authors have attempted to explain the pathophysiology of NAFLD by advancing the “multiple parallel hits hypothesis” [5]. However, the specific process responsible for the development and progression of NAFLD is still an open question. While SS is considered a relatively benign condition with little risk of progression, NASH may progress to cirrhosis and, in a small percentage of patients, to hepatocellular carcinoma (HCC) [6]. In fact, there is increasing evidence to indicate a complex interplay between environmental genetic factors that predispose the progression of NAFLD [7].

Patatin-like phospholipase domain-containing protein 3 (PNPLA3), which is also known as adiponutrin, is mainly expressed in hepatocytes but also in adipocytes [8]. The protein is one of the candidates potentially related to NAFLD susceptibility. Regarding PNPLA3 lipase activity against TG and acylglycerol transacetylase activity, its expression is responsible for energy mobilization and the storage in lipid droplets [9,10]. Additionally, it is highly modulated by nutritional stimuli at transcriptional and posttranscriptional levels [11].

In 2008, Romeo *et al.* [12] reported that a *PNPLA3* single nucleotide polymorphism at residue 148 in the DNA sequence, resulting in a substitution of isoleucine for methionine (I148M, rs738409), was a genetic determinant of NAFLD. Since then, the correlation between the *PNPLA3* 148M variant and NAFLD has been investigated in considerable detail. Multiple studies have demonstrated a link between the *PNPLA3* 148M variant and the development and progression of NAFLD, including liver fibrosis [13,14,15,16,17,18]. Recently, it has been reported that *PNPLA3* 148M elevates retinyl-palmitate content in human hepatic stellate cells providing evidence for a potential link between the PNPLA3 variant, human hepatic retinoid metabolism, and chronic liver disease [19,20]. All this research indicates that this variant is a potential modifier of NAFLD. Nevertheless, its role in the NAFLD development and the specific molecular mechanisms has not been fully elucidated.

Lipid deposition in the liver seems to be a critical mechanism in the pathogenesis of NAFLD, so its regulatory processes need to be elucidated if the progression of NAFLD is to be controlled. Although these potential regulatory mechanisms are multiple, one of them affecting TG remodeling could be PNPLA3 [21,22,23].

On the basis of this data, the aim of our work was to study the relationship between the liver expression of *PNPLA3* and the presence of NAFLD in morbidly obese women. Furthermore, as lipid metabolism seems to be involved in the pathogenesis of NAFLD, we investigated the association between the hepatic expression of *PNPLA3* and the expression of the main lipid metabolism-related genes. Finally, in order to explore the impact of the *PNPLA3* genetic variant on the presence of NAFLD, we determined the relationships between the rs738409 polymorphism in the *PNPLA3* gene and the severity of the disease.

## 2. Results

### 2.1. General Characteristics of Cohort

Our morbidly obese women (MO) cohort was sub-classified according to liver pathology study into normal liver (NL, *n* = 18), simple steatosis (SS, *n* = 20), and non-alcoholic steatohepatitis (NASH, *n* = 17) (Table 1). We found no significant differences regarding age and anthropometrical measurements between the three groups studied. With regard to biochemical analysis, glucose levels were significantly increased in the SS and NASH groups compared to the NL group (*p* = 0.017 and *p* = 0.010). Glycosylated hemoglobin (HbA1c) levels were also higher in SS than in NL (*p* = 0.039). Our results showed that aspartate aminotransferase (AST) and alanine aminotransferase (ALT) activity were higher in the NASH group than in the NL group (*p* = 0.001 and *p* ≤ 0.001) and that ALT was increased in NASH compared to SS (*p* = 0.001).

### 2.2. Determination of Patatin-Like Phospholipase Domain-Containing Protein 3 (PNPLA3) Liver Expression

**Figure 1:**
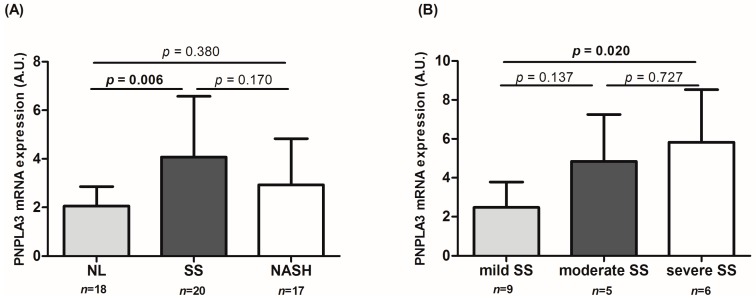
Hepatic expression of *PNPLA3* gene in morbidly obese women according to the liver histopathology (**A**) and subclassifying the SS group into: mild, moderate, or severe SS (**B**). A.U.: arbitrary units; NASH: morbidly obese women with steatohepatitis; NL: morbidly obese women with a normal liver; SS: morbidly obese women with simple steatosis. ANOVA test was used to determinate differences between groups. *p* < 0.05 are considered statistically significant.

We analyzed *PNPLA3* liver expression in MO women in relation to the presence of NAFLD. The results showed that *PNPLA3* expression was a significant 72% greater in MO NAFLD women than in MO women with NL (MO NAFLD: 3.6 ± 2.2 and MO NL: 2.1 ± 0.8, *p* = 0.001). Furthermore, when we classified the MO cohort into NL, SS, and NASH, we observed that the expression of *PNPLA3* was significantly higher in SS than in NL (*p* = 0.006, Figure 1A). There were no differences between NL or SS and NASH (*p* = 0.380 and *p* = 0.170, respectively). It is important to note that, in our work, any patient with steatohepatitis had fibrosis in the liver histology, so we could not perform correlations between fibrosis staging and *PNPLA3* liver expression.

In addition, in order to explore the increased expression of *PNPLA3* in simple steatosis, we classified the SS group into grades: mild (*n* = 9), moderate (*n* = 5), or severe SS (*n* = 6). We found that the expression of *PNPLA3* was significantly increased in the severe group compared to the mild SS group (*p* = 0.020, Figure 1B).

### 2.3. Correlations between the Expression of PNPLA3 and Biochemical Variables, Histopathological Parameters and Genes Involved in Lipid Metabolism and Inflammation in Liver from Morbidly Obese Subjects

When we analyzed the associations between *PNPLA3* expression and parameters related to glucose metabolism and lipid profile, we observed a direct correlation between circulating levels of triglycerides and *PNPLA3* expression in the whole study cohort (*r* = 0.272, *p* = 0.046).

Regarding histopathological features, we only found a direct association between *PNPLA3* expression and degree of steatosis in the total MO group (*r* = 0.441, *p* = 0.001).

In order to clarify whether *PNPLA3* was associated with hepatic lipid metabolism, we studied the correlation between *PNPLA3* expression and lipid metabolism related genes in liver from the MO cohort. In the lipogenic and fatty acid oxidation pathways, hepatic liver X receptor (*LXRα*) and peroxisome-proliferator-activated receptor α (*PPARα*) expression correlated directly with PNPLA3 expression in the total morbidly obese group (*r* = 0.671, *p* = 0.008 and *r* = 0.640, *p* = 0.008; Table 2). We also showed a positive association between *PNPLA3* and both the transcription factor sterol regulatory element binding protein 2 (*SREBP2*) (*r* = 0.412, *p* = 0.032) and lipocalin 2 (*LCN2*) (*r* = 0.570, *p* = 0.032) in the whole population.

Interestingly, when we analyzed the relationship between the expression of these genes in the SS subgroup, we observed that both *LXRα* and *PPARα* correlations were stronger (*LXRα*: *r* = 0.806, *p* = 0.016; *PPARα*: *r* = 0.796, *p* = 0.024).

### 2.4. rs738409 Genotype Distribution in Morbidly Obese Subjects

The distribution of the studied genetic polymorphism is shown in Table 3, as are comparisons between NL, SS, and NASH patients. The G allele was more frequent (66.6%) than the C allele (33.3%) in the whole population. No individuals were homozygous for the C allele. The genotype frequencies of the rs738409 polymorphism showed significant variations between NL, SS, and NASH patients (*p* = 0.021). In addition, the GG genotype was correlated with the presence of NASH (*r* = 0.382, *p* < 0.001). However, the allele frequencies did not show statistically significant differences (*p* = 0.145). Regarding clinical and biochemical variables, the GG genotype was only associated with increased body mass index (BMI) (*r* = 0.300, *p* = 0.032). There was no association between PNPLA3 genetic variant and its hepatic expression (*p* = 0.478).

## 3. Discussion

In an own previous work, we demonstrated a downregulation of the lipogenic pathway related to the severity of steatosis in a cohort of women with morbid obesity [25]. As PNPLA3 seems to be related with the accumulation of hepatic TG, in the present study, we examined the relationship between the liver expression levels of *PNPLA3*, the key lipid metabolism-related genes expression, and the clinicopathological factors in a cohort of morbidly obese women with NAFLD. In our study, 36% and 31% of morbidly obese women were diagnosed with SS and NASH, respectively, using the diagnostic *gold standard* liver biopsy. Our findings show that *PNPLA3* liver expression was increased in morbidly obese women with NAFLD. It is important to note that we have demonstrated a clear relationship between *PNPLA3* and the degrees of SS, suggesting a direct correlation between *PNPLA3* and the severity of steatosis.

Nowadays, more than 50 studies on the genotyping of *PNPLA3* have confirmed the association between the 148M variant and the full range of NAFLD, including simple steatosis, steatohepatitis, cirrhosis, and hepatocellular carcinoma. PNPLA3 148M has been shown to be related to an increased risk of NAFLD across multiple ethnic groups [26,27,28,29,30,31,32,33,34]. The aim of the present work was to compare the hepatic expression of *PNPLA3* in a cohort of morbidly obese women presenting a normal liver or NAFLD. We showed that the hepatic expression of PNPLA3 in morbidly obese women with NAFLD was higher than in MO women with NL. Consistent with our work, Kotronen *et al.* [8] described a direct correlation between *PNPLA3* liver expression and liver fat content measured by magnetic resonance. It is important to note that our study confirms this finding in biopsy-proven NAFLD. Regarding steatosis degree, recent studies observed that a variant of this protein has an association with moderate-to-severe steatosis [35,36]. Although these studies analyzed only a variant of PNPLA3, not its liver expression, their results are in agreement with ours. A recent interesting work by Donati *et al.* [37] has demonstrated that PNPLA3 rs2294918 E434K diminished *PNPLA3* expression and protein levels, lessening the effect of the rs738409 polymorphism on the predisposition to steatosis liver injury. Moreover, the authors suggested that this PNPLA3 variant had a codominant negative effect on TG mobilization from lipid droplets. Regarding non-alcoholic steatohepatitis, a DNA microarray study in human liver revealed an upregulation of *PNPLA3* in NASH *vs.* healthy controls [38]. Nevertheless, Kitamoto *et al.* [39] described lower *PNPLA3* mRNA levels in the liver of patients with an advanced grade of NAFLD (with fibrosis) compared with those with mild NAFLD. However, we were not able to reproduce any of these findings. Perhaps the differences in the groups studied in these works regarding age, gender, BMI, or race can explain these discrepancies.

Because PNPLA3 has previously been reported to influence lipid metabolism in animal models and in *in vitro* studies [40,41], we evaluated the interplay of *PNPLA3* liver expression with the expression of the main lipid metabolism-related genes. In the current first human study in this sense, *PNPLA3* expression positively correlated with *LXRα*, *PPARα*, and *SREBP2* liver expression. All these proteins are transcription factors that relate to response elements found in a various genes that are associated with lipid turnover including their own genes [42]. Specifically, *LXRα* belong to the nuclear hormone receptor superfamily of ligand-activated transcription factors as *SREBP1* which, in liver, serve as lipid sensors and regulate the expression of main genes which modulate the cholesterol and FA metabolism [43]. Regarding NAFLD, interaction between *LXR* and *SREBP1* is a crucial step in the molecular cascade of events characterizing steatogenesis [44]. In this regard, Huang *et al.* [41] determined that the overexpression of the three SREBP family members (*SREBP1a*, *1c*, and *2*) increases liver *PNPLA3* expression in mice. They also found that PNPLA3 expression was regulated by *SREBP1c* and *LXRα*. Similar results were described by Dubuquoy *et al.* [45], who showed that, in the mouse liver, *PNPLA3* gene expression was under the direct transcriptional control of *SREBP1c* in response to insulin. However, at variance with murine studies, we were not able to find any association with *SREBP1*, one of the key genes related to *de novo* lipogenesis. Moreover, Mancina *et al.* [34] conducted a study to evaluate the contribution of *de novo* lipogenesis to liver fat accumulation in the PNPLA3 I148M genetic variant of NAFLD. They showed a dissociation between hepatic *de novo* lipogenesis and liver fat content due to the PNPLA3 148M allele, suggesting that increased *de novo* lipogenesis is not a main feature in all subjects with steatosis. However, these authors have not studied the hepatic expression of *PNPLA3*. Regarding the positive relationship between *PPARα* and PNPLA3, it is known that *PPARα* seems to control the expression of genes regulating peroxisomal/mitochondrial β-oxidation [46]. In this context, perhaps the induction of fatty acid catabolism might act as a defense mechanism, preventing hepatocellular fat accumulation [47]; in other words, it might represent an inefficient physiological response to counteract steatosis by promoting the β-oxidation of fatty acids in the hepatocytes. In our study, the association between *PNPLA3* and *SREBP2* may suggest a novel association with cholesterol metabolism in humans. Currently, experimental and human evidence has related to altered hepatic cholesterol metabolism and free cholesterol accumulation to the pathogenesis of steatosis and liver damage [48]. Specifically, Min *et al.* [49] have demonstrated dysregulated cholesterol metabolism in NAFLD, which may contribute to disease severity through activation of SREBP2 and 3-Hydroxy-3-Methylglutaryl-CoA Reductase (HMGCR).

In the present work, we observed an interesting association between liver PNPLA3 expression and the liver expression of *LCN2* in the severely obese women group, which has not been previously described. In one of our previous studies, we described increased liver *LCN2* expression in NAFLD, and this expression positively correlated with SS [50]. Additionally, in this work, an increased regulation of *LCN2* expression was detected in *in vitro* experiments with HepG2 cells under harmful conditions. Perhaps, as some authors have suggested, *LCN2* is a protective molecule [51]—in this case, against the development of NAFLD.

Moreover, we did not find any relationship between *PNPLA3* liver expression and other adipocytokines studied, probably because the molecular function of PNPLA3 is related to cellular lipid accumulation in the liver more than with inflammation [52]. Unexpectedly, we did not find any relationship with the expression of genes related to transport and the uptake of fatty acids. Perhaps this mechanism of liver accumulation of fatty acids has a lower contribution in humans, as we and other authors have previously shown [25,38].

Finally, to explore the effect of the *PNPLA3* genetic variant with a potential impact on NAFLD, we determined the relationship between the rs738409 polymorphism in the *PNPLA3* gene and the severity of disease. In this sense, we found that the GG genotype, encoding I148M, was directly correlated with the presence of NASH. Our results are similar to recent studies that showed a relationship between this genetic variant and the severity of NAFLD [12,13,15,53]. Consistent with our results, Kotronen *et al.* [8] observed that there were no differences in the hepatic *PNPLA3* mRNA expression between different *PNPLA3* genotype carriers.

We should point out the following drawbacks of our study. The main limitation of this work is an adjusted sample size and the lack of evaluation of protein expression. Additionally, the study is cross-sectional. We could not prove a causal link between *PNPLA3* expression and NALFD development. However, our study cohort of morbidly obese women has revealed clear relationships between the expression of *PNPLA3* and NAFLD, without the interference of gender or age. Thus, our findings cannot be extrapolated to men or other obesity groups such as normal-weight or over-weight women.

## 4. Materials and Methods

### 4.1. Subjects

The study was approved by the ethics committee of the Hospital Joan XXIII (23c/2015, Tarragona, Spain), and all subjects gave written informed consent. We included 55 Caucasian MO women (BMI > 40 kg/m^2^). Liver biopsies were obtained during planned laparoscopic bariatric surgery and were performed for clinical indications.

The diagnosis of NAFLD was made using the following criteria: (1) liver pathology; (2) an intake of less than 10 g of ethanol/day; and (3) appropriate exclusion of other liver diseases.

The body weight of all women had not fluctuated more than 2% for at least 3 months prior to bariatric surgery. The exclusion criteria were: (1) concurrent use of medications known to produce hepatic steatosis; (2) patients using hypolipemiant treatment; (3) diabetic subjects who were receiving insulin or on medication likely to influence endogenous insulin levels; (4) menopausal or post-menopausal women; (5) women undergoing contraceptive treatment and subjects receiving contraceptive treatment; (6) patients who had an acute illness, current evidence of acute or chronic inflammatory or infectious diseases, or malignant diseases.

### 4.2. Liver Pathology

Liver samples were processed by two experienced hepatopathologists using methods previously described [54,55]. Simple steatosis (SS) was graded as follows: Grade 1 or mild SS: more than 5% and less than 33% of hepatocytes affected; Grade 2 or moderate SS: 33% to 66% of hepatocytes affected; or Grade 3 or severe SS: more than 66% of hepatocytes affected. Moreover, the minimum criteria for the steatohepatitis diagnosis included the presence of either ballooning cells and lobular inflammation or perisinusoidal/pericellular fibrosis in zone 3 of the hepatic acinus.

According to liver pathology, women were sub-classified into: (1) normal liver (NL) histology (*n* = 18); (2) simple steatosis (SS) (micro/macrovesicular steatosis without inflammation or fibrosis, *n* = 20); (3) non-alcoholic steatohepatitis (NASH) (Brunt Grades 1–3, *n* = 17).

### 4.3. Biochemical Analyses

Each of our patients was evaluated with a complete physical, anthropometrical, and biochemical assessment. BMI was calculated as body weight divided by height squared (kg/m^2^). Fasting glucose, insulin, HbA1c, HDL-C, LDL-C, TG, and transaminases were measured using a conventional automated analyzer after overnight fasting. Insulin resistance was calculated using HOMA2-IR [56].

### 4.4. RNA Isolation and Real-Time PCR

Liver samples were preserved in RNAlater (Sigma, Barcelona, Spain) for 24 h at 4 °C and then stored at −80 °C. Total RNA was extracted by using an RNeasy mini kit (Qiagen, Barcelona, Spain). And was reverse transcribed by the High Capacity RNA-to-cDNA Kit (Applied Biosystems, Madrid, Spain). Real-time quantitative PCR was carried out with the TaqMan Assay predesigned by Applied Biosystems for the detection of *PNPLA3* (Hs00228747_m1), *ABCA1* (Hs01059118_m1), *ABCG1* (Hs00245154_m1), *ADIPOR2* (Hs00226105_m1), *ACC1* (Hs00167385_m1), *CROT* (Hs00221733_m1), *FABP4* (Hs00609791_m1), *FAS* (Hs00188012_m1), *IL6* (Hs00985639_m1), *LCN2* (Hs00194353_m1), *LXRα* (Hs00173195_m1), *PPARα* (Hs00947538_m1), *RESISTIN* (Hs00220767_m1), *SREBP1c* (Hs01088691_m1), *SREBP2* (Hs01081784_m1), *TNFα* (Hs99999043_m1), and *18S ribosomal RNA* (4352930E), which was used as the housekeeping gene. All reactions were performed in duplicate using the 7900HT Fast Real-Time PCR systems (Applied Biosystems).

### 4.5. Genotyping

Subjects were genotyped for the rs738409 polymorphism using the TaqMan 5′ allelic discrimination assay (TaqMan SNP Genotyping Assay C 7241 10, Applied Biosystems,). Amplifications were carried out using the 7900HT Sequencing Detection System for continuous fluorescence monitoring.

### 4.6. Statistical Analysis

We used the SPSS/PC+ statistical package for Windows (version 22.0; SPSS, Chicago, IL, USA). One-way ANOVA with a *post-hoc* Tukey test was carried out to determine differences between groups. The correlations between variables was analyzed using Pearson’s method (parametric variables) and Spearman’s test (non-parametric variables). Allele and genotype frequencies were evaluated with the χ-squared test. *p*-Values <0.05 were considered to be statistically significant.

## 5. Conclusions

The main results of our study show that liver *PNPLA3* expression is increased in NAFLD patients and is particularly associated to severity of steatosis. Moreover, *PNPLA3* expression is correlated with the expression of main cholesterol and hepatic lipid metabolism-related genes. Further human studies are required to confirm these associations.

## Figures and Tables

**Table 1:** General characteristics of the studied cohort classified according to the liver pathology.

Variables	Morbidly Obese Subjects (*n* = 55)		
	NL (*n* = 18)	SS (*n* = 20)	NASH (*n* = 17)
	Mean ± SD	Mean ± SD	Mean ± SD
Age (years)	48.6 ± 10.9	50.4 ± 11.0	47.8 ± 13.0
Weight (kg)	120.5 ± 19.3	120.4 ± 18.1	116.6 ± 15.5
BMI (kg/m^2^)	50.1 ± 7.6	48.8 ± 8.5	47.0 ± 4.8
WC (cm)	130.0 ± 17.9	129.5 ± 12.9	129.4 ± 12.0
Glucose (mg/dL)	94.2 ± 22.6	133.9 ± 50.6 *	138.7 ± 49.1 *
Insulin (mUI/L)	12.1 ± 7.8	18.6 ± 12.3	20.1 ± 16.4
HbA1c (%)	5.2 ± 0.9	6.5 ± 1.7 *	6.3 ± 1.6
HOMA2-IR	1.6 ± 0.9	2.8 ± 1.4	2.8 ± 2.1
HDL-C (mg/dL)	44.5 ± 9.8	36.8 ± 11.3	37.1 ± 5.9
LDL-C (mg/dL)	99.0 ± 27.3	100.9 ± 29.3	104.4 ± 31.2
Total cholesterol (mg/dL)	173.03 ± 35.53	169.55 ± 34.04	174.81 ± 33.66
Triglycerides (mg/dL)	136.5 ± 58.4	193.1 ± 128.6	174.0 ± 81.1
AST (U/L)	23.5 ± 12.3	40.2 ± 33.9	64.9 ± 35.8 *
ALT (U/L)	22.1 ± 8.5	37.6 ± 22.9	67.0 ± 33.4 *^,#^
GGT (U/L)	26.6 ± 23.5	27.6 ± 14.8	53.7 ± 59.5
ALP (U/L)	61.9 ± 12.4	74.1 ± 20.3	79.9 ± 29.7

ALP: alkaline phosphatase; ALT: alanine aminotransferase; AST: aspartate aminotransferase; BMI: body mass index; GGT: gamma-glutamyltransferase; HbA1c: glycosylated hemoglobin; HDL-C: high density lipoprotein cholesterol; HOMA2-IR: homeostatic model assessment 2-insulin resistance; LDL-C: low density lipoprotein cholesterol; NASH: morbidly obese subjects with steatohepatitis; NL: morbidly obese subjects with normal liver; SS: morbidly obese subjects with simple steatosis; WC: waist circumference. One-way ANOVA with *post-hoc* Tukey test was used to compare variables between groups. * indicates statistically significant differences respect NL group (*p* < 0.05); # indicates statistically significant differences respect SS group (*p* < 0.05). Data are expressed as mean ± SD.

**Table 2:** Correlations between *PNPLA3* expression and genes related to *de novo* lipogenesis, FA oxidation, FA transport and uptake, inflammation, adipocytokines, and cholesterol metabolism in livers from MO women and those sub-classified as SS in the MO cohort.

Variables	MO PNPLA3 (*n* = 55)		SS PNPLA3 (*n* = 20)	
	*r*	*p*-Value *	*r*	*p*-Value *
*De Novo Lipogenesis*				
*SREBP1c*	−0.016	0.920	0.130	0.906
*LxRα*	**0.671**	**0.008**	**0.806**	**0.016**
*ACC1*	−0.025	0.920	0.090	0.906
*FAS*	−0.021	0.920	0.114	0.906
*Fatty Acid Oxidation*				
*PPARα*	**0.640**	**0.008**	**0.796**	**0.024**
*CPT1α*	0.134	0.576	−0.233	0.906
*CROT*	0.200	0.466	0.098	0.906
*Cholesterol Metabolism*				
*ABCA1*	0.016	0.920	−0.189	0.906
*SREBP2*	**0.412**	**0.032**	0.361	0.784
*Transport and Uptake FA*				
*FABP4*	−0.371	0.285	0.464	0.784
*ABCG1*	0.099	0.713	−0.074	0.906
*Inflammation*				
*IL6*	−0.379	0.285	−0.012	0.980
*TNFα*	0.227	0.576	0.089	0.906
*LCN2*	**0.570**	**0.032**	0.466	0.784
*Adipokines*				
*RESISTIN*	0.209	0.576	0.124	0.906
*ADIPOR2*	−0.245	0.576	0.491	0.784

ABCA1: ATP-binding cassette transporter A1; ABCG1: ATP-binding cassette transporter G1; ADIPOR2: adiponectin receptor; ACC1: acetyl-coenzyme A carboxylase 1; CROT: carnitine O-octanoyltransferase ; FA: fatty acid; FABP4: fatty acid binding protein 4; FAS: fatty acid synthase; IL6: interleukin 6; LCN2: lipocalin 2; LXRα: liver X receptor; MO: morbidly obese women; PPARα: peroxisome-proliferator-activated receptor α; SREBP1c: sterol regulatory element binding protein 1c; SREBP2: sterol regulatory element binding protein 2; SS: simple steatosis; TNFα: tumor necrosis factor. Bold numbers indicate statistically significant correlations (*p*-value < 0.05). * *p*-Value adjusted by the Benjamini–Hochberg method [24].

**Table 3:** The distribution of rs738409 polymorphism in morbidly obese women according to liver histology.

Groups	Genotype, *n* (%)		Allele, *n* (%)	
	CG	GG	C	G
NL (*n* = 16)	12 (75)	4 (25)	12 (37.5)	20 (62.5)
SS (*n* = 18)	15 (83.3)	3 (16.6)	15 (41.7)	21 (58.3)
NASH (*n* = 17)	7 (41.2)	10 (58.8)	7 (20.6)	27 (79.4)

CG: individuals carrying the genotype (CG); GG: individuals carrying the genotype (GG); C: Allele C; G: Allele G; NASH: morbidly obese subjects with steatohepatitis; NL: morbidly obese subjects with normal liver; SS: morbidly obese subjects with simple steatosis.

## Abbreviations

ABCA1	ATP-binding cassette transporter A1
ABCG1	ATP-binding cassette transporter G1
ADIPOR	adiponectin receptor
ACC1	acetyl-coenzyme A carboxylase 1
ALT	alanine aminotransaminase
AST	aspartate aminotransaminase
ALP	alkaline phosphatase
BMI	body mass index
CROT	carnitine *O*-octanoyltransferase
FABP4	fatty acid binding protein 4
FAS	fatty acid synthase
18S	18S ribosomal RNA
GGT	γ-glutamyl transferase
HbA1c	glycosylated hemoglobin
HDL-C	high density lipoprotein
HOMA2-IR	homeostasis model assessment of insulin resistance
IL6	interleukin 6
LDL-C	low density lipoprotein
LCN2	lipocalin 2
LXRα	liver X receptor
MO	morbidly obese
NAFLD	non-alcoholic fatty liver disease
NASH	non-alcoholic steatosis
NL	normal liver
PPARα	peroxisome-proliferator-activated receptor α
SREBP1c	sterol regulatory element binding protein 1c
SREBP2	sterol regulatory element binding protein 2
SS	simple steatosis
TG	triglycerides
TNFα	tumor necrosis factor
WC	waist circumference
